# Saying ‘no’ with confidence: statistical approaches to test for the absence of an effect

**DOI:** 10.1098/rsbl.2025.0506

**Published:** 2025-10-29

**Authors:** Lewis G. Halsey

**Affiliations:** ^1^ School of Life and Health Sciences, University of Roehampton, London, UK

**Keywords:** Bayes factors, Bayesian estimations, confidence intervals, equivalence testing, likelihood ratios, null hypothesis significance testing

## Abstract

Publishing non-significant findings is essential for the progress of science. However, many of us forget that ‘absence of evidence is not evidence of absence’ and believe that a statistically non-significant result is evidence of no effect. Regrettably, and despite the null hypothesis being simple, elegant and often underpinned by evidenced or reasoned convictions, conventional *p*-value analysis can only argue against the null hypothesis, never in favour of it. Here, I provide a quick-and-easy guide to simple yet powerful statistical options available to biologists for investigating the absence of a meaningful effect, namely equivalence tests, confidence intervals and credible intervals; or the absence of any effect, namely likelihood ratios and Bayes factors. These approaches, supported by accessible software, allow biologists to draw direct conclusions about the null hypothesis.

Biologists strive for ‘significant results’. We want to discover correlations between traits, differences between experimental conditions and associations between treatments and outcomes. At the same time, most of us are aware that it is also valuable to understand when traits do not relate and when perturbations are not effective [1,2]. For example, maybe it is interesting to know if two chemical interventions to reduce crop pestilence work equally well, given that one is far more toxic and expensive.

But many of us do not realize that a non-significant statistical result is not stating that there is no effect [3]. It is wrong to claim ‘there is no correlation’ or ‘there is no difference’ when the *p*-value is high. Fisher himself made clear that a *p*-value does not provide evidence for the null hypothesis, regardless of how ‘non-significant’ it is [4]; in such cases, we can only conclude that we find our data unsurprising if we believe in the null hypothesis.

Frequentist statistics are the most common form of inferential statistics used in the biological sciences and are almost always applied within the context of a null hypothesis of no effect. The null hypothesis is assumed to be true, and the analysis assesses the probability of observing the sampled data on this basis—if the probability is sufficiently low, then we might reasonably conclude there is, in fact, an effect (cf. [5]). We cannot, however, simply reverse this concept and conclude from a relatively high *p*-value that there is not an effect. Put simply, because the *p*-value assumes the null hypothesis is true, it cannot therefore evaluate the likelihood that this is so. The only safe way to report a high *p*-value is ‘there was no statistically significant correlation between X and Y’ or ‘no statistically significant difference between P and Q’, which is not providing any interpretation beyond the observation that *p* is greater than the threshold for statistical significance.

Unfortunately, around half of scientific research papers falsely report non-significant results as indicating no effect [3]. For any field of biology, this creates a warped picture of the state of the art. Moreover, even when non-significant results are interpreted accurately, they often seem unsatisfactory because we cannot interpret whether our result is due to the lack of an effect or the lack of necessary power in our study to find the effect.

There are, however, methods available to us for viably concluding no effect based on our data. Here, I outline several statistical options for this task, starting with the option associated with the statistics we are probably most familiar with, that of null hypothesis significance testing.

## 1. Test of equivalence: is there sufficient evidence to reasonably conclude that the null hypothesis of no meaningful effect is true?

With the exception of count data, an effect size is very rarely if ever absolutely zero. In turn, it is not straightforward to conclude there is no effect because the data always offer some level of evidence otherwise, i.e. *p* is almost always less than 1 [6]. In recognition of this, Hodges & Lehmann wrote the seminal work on an approach that applies null hypothesis significance testing for no effect; specifically, no meaningful effect. They considered the issue in terms of comparing the means of two groups, and proposed testing whether the two means do not differ by more than a predetermined smallest effect size of importance. In other words, rather than testing against a nil null hypothesis (i.e. where the null is 0), you test against a non-nil null hypothesis(!) (i.e. where the null is something other than 0). From this concept was born non-equivalence tests, which entail two single-tailed tests of (i) whether the treatment mean is smaller than a predetermined largest effect size of importance and (ii) larger than a lowest effect size [7]. If both tests are statistically significant, then you can reject the two non-nil null hypotheses and conclude that the treatment effect is smaller than the smallest meaningful effect size. Equivalence tests are not immediately intuitive; a visual example will clarify.

**Figure 1.**
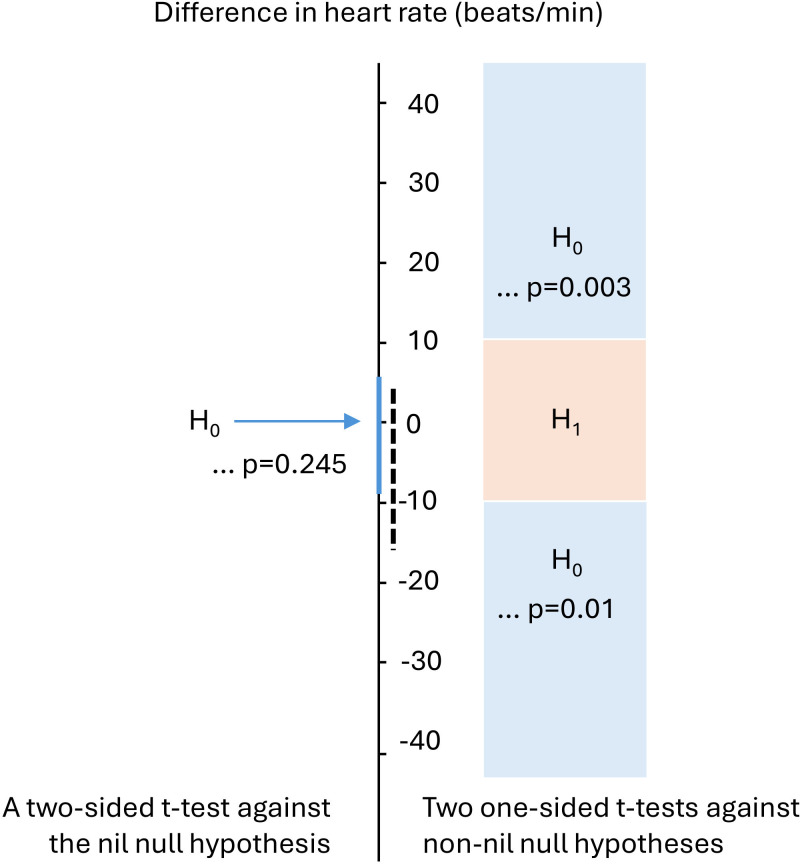
An equivalence test for differences in the hypothetical heart rate measurements (in beats min^−1^) of deer during two behaviours: walking and rutting. The test comprises two one-sided *t*-tests of null hypotheses: the difference is greater than 10 beats min^−1^ and the difference is less than −10 beats min^−1^. The *p*-values associated with each of these *t*-tests are displayed (see also main text). The 90% confidence interval associated with the sample data is presented as the vertical solid blue line and indicates that values more extreme than −10 or 10 beats min^−1^ can be rejected, and there is equivalence, i.e. evidence of no meaningful effect. The dashed vertical line represents a 90% confidence interval in a scenario where you cannot reject the reasonable possibilities that either the difference in heart rate between the two conditions is meaningful or that it is 0 (see main text). The annotation to the left of the vertical axis denotes the point nil null hypothesis tested against using a two-sided *t*‐test.

Imagine you want to test whether the heart rate of male red deer does not differ between periods of walking and of rutting, and you *a priori* decide, based on heart rate–metabolic rate equations, that a difference (in either direction) of anything less than 10 beats min^−1^ is not meaningful because this represents the magnitude of the error associated with the metabolic rate estimates (figure 1). A two-sided *t*‐test would return *p* = 0.245; however, you are instead undertaking a test for equivalence and thus conduct two one-sided *t*-tests. With the first of these one-sided *t*-tests you test whether the mean difference in heart rate is less than 10 beats min^−1^: *p* = 0.003 and so, taking the Neyman–Pearson approach to statistical inferences [8], you reject the non-nil null that the difference is larger than 10 beats min^−1^. With the second one-sided *t*‐test, you test whether the mean difference is a value higher than −10 beats min^−1^: *p* = 0.01 and so you can also reject the non-nil null of a difference absolutely greater than −10 beats min^−1^. (Thinking about a negative difference when assessing the second one-sided *t*‐test adds a bit more cognitive load to interpreting an equivalence test, but do persevere and keep referring to figure 1.) Overall, then, although you cannot conclude that the heart rate difference between the two behaviours is 0, you can conclude that the heart rate difference is less than 10 beats min^−1^ and thus lower than any meaningful difference.

Here is an example of when you cannot reasonably draw the conclusion that there is no (meaningful) difference in red deer heart rate while walking versus rutting—you conduct two one-sided *t*-tests with non-nil null hypotheses of 10 and −10 beats min^−1^, which return *p* = 0.003 and *p* = 0.437, respectively. So while again you can reject the non-nil null that the difference is larger than 10 beats min^−1^, this time you cannot reject the non-nil null of a difference absolutely greater than −10 beats min^−1^. Overall, then, you cannot reject the reasonable possibility that the difference in heart rate between the two conditions is meaningful; the data are inconclusive and you must collect more to obtain a conclusive answer. This interpretation contrasts the spurious claim many researchers would make based on the two-sided *t*‐test (*p* = 0.245) that there is no difference in heart rate between the conditions. You need to collect more data to obtain a conclusive answer.

Equivalence tests can also be undertaken for correlations where, similarly to one-sided *t*-tests potentially rejecting non-nil null hypotheses of absolute values larger than a prespecified threshold, one-sided correlations potentially reject correlation coefficient values more extreme than a threshold.

The use of equivalence tests in the life sciences is rare; why? I suspect because few students of statistics are made aware of such tests, and historically at least, equivalence tests were not incorporated into most statistics packages. However, now equivalence tests for differences, correlations, proportions and meta-analyses can be undertaken using the TOSTER package in R [9,10], or a pre-formatted Excel spreadsheet [10], or using the free software packages Jamovi or JASP.

## 2. Confidence interval bound: is there sufficient evidence to reasonably conclude that the nil null hypothesis of no meaningful effect is true?

An assessment of confidence intervals can match undertaking equivalence tests. (NB, because the equivalence test is based on two one-sided tests, a 90% confidence interval is appropriate when those tests are assessed against the 5% alpha level [11, section 9.6].) Tracking the two scenarios depicted in §1, if the confidence interval includes 0 but does not include either 10 or −10 beats min^−1^, then while you cannot reject the null hypothesis of no difference, you can conclude that the heart rate difference is smaller than any meaningful difference (figure 1). Alternatively, if the confidence interval includes 0 and either 10 and/or −10 beats min^−1^, then you have again failed to reject the null hypothesis of no difference, but this time also the non-nil null hypothesis of the smallest effect size of interest [12] (figure 1). (Note that a confidence interval that is within the bound of −10 and 10 beats min^−1^, but which does not include the values of 0, or 10 or −10, should draw the conclusion that the difference in heart rate between behaviours is neither 0 nor meaningful.)

## 3. Likelihood ratio: based on the current data, what is the evidence for the nil null hypothesis compared to a stated precise alternative hypothesis?

Many a criticism has been made of the so-called ‘dichotomania’ [3] that typically accompanies *p*-value analyses [6,13–15]. Pertinent to the current article is the concern that the approaches in §§1 and 2 both support decisions conditional on a null hypothesis rather than as a measure of support conditional on the observed data [16]. A fundamentally different way to evaluate the absence of an effect is to consider how much evidence your data offers in support of the null hypothesis (usually a nil null hypothesis) compared to an alternative hypothesis (of an effect of stated magnitude). This is the likelihood ratio; specifically, quantifying the relative likelihood of the data you have observed in the scenario of the nil null hypothesis and a stated alternative hypothesis, where both those scenarios are represented as point values.

**Figure 2.**
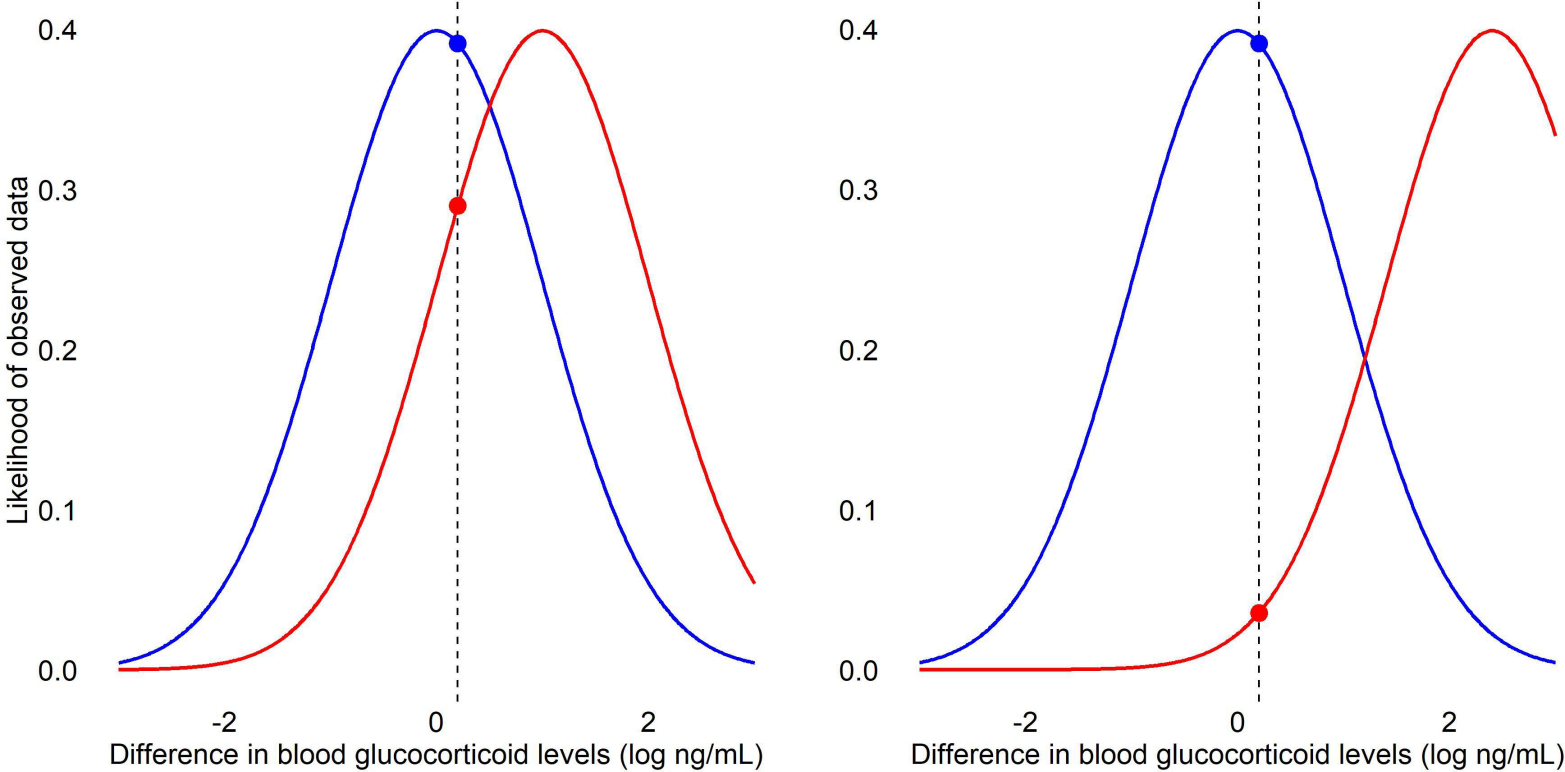
Likelihood ratio tests of differences in the hypothetical blood glucocorticoid levels of laboratory-housed zebra finches chronically exposed to different aviary configurations. The likelihood curves indicate the likelihood of the observed data under the nil null hypothesis (blue) and the alternative hypothesis of a stated difference (red). The vertical dashed line marks the observed mean difference. The circles marking the intercept of the dashed line and likelihood curves represent the likelihood values under the two hypotheses for the observed difference. In the left-hand panel, the alternative hypothesis is a difference of log 1 ng ml^−1^ and the observed difference is log 0.2 ng ml^−1^. The likelihood value for the null is 0.39 and for the alternative is 0.29, resulting in a likelihood ratio of 1.35. In the right-hand panel, while the observed difference is again log 0.2 ng ml^−1^, the alternative hypothesis is a difference of log 2.3 ng ml^−1^. The likelihood value for the null is again 0.39 but for the alternative it is 0.04, resulting in a likelihood ratio of 11.02.

For example, glucocorticoid levels in birds can vary greatly [17]; let us consider a hypothetical study of the blood glucocorticoid levels of laboratory-housed zebra finches chronically exposed to different aviary configurations. The ratio calculated from a likelihood test of the observed data is 1.35, indicating that the data are 1.35 times more likely under the null hypothesis of no difference than under the alternative hypothesis of some pre-stated level of difference (assuming the null is the numerator in the ratio) (figure 2). While the likelihood ratio necessary to conclude that the nil null hypothesis can be believed is subjective, Royall [18] proposes that a ratio of 8 be classed as moderately strong evidence in favour of the nil null and four times more than this (i.e. 32) as strong evidence. It is important to bear in mind, however, that likelihood ratios only compare the hypotheses provided; other hypotheses may be even more plausible given the observed data. You can apply a likelihood ratio test to your data manually using the package lmtest() in R [19]; care should be taken when likelihood ratios are provided by default in association with linear models because these are usually maximum likelihood calculations where the alternative hypothesis has been cherry-picked from all possible candidate values.

## 4. Bayes factor: based on the current data, what is the evidence for the nil null hypothesis compared to a stated distribution of alternative hypotheses?

**Figure 3.**
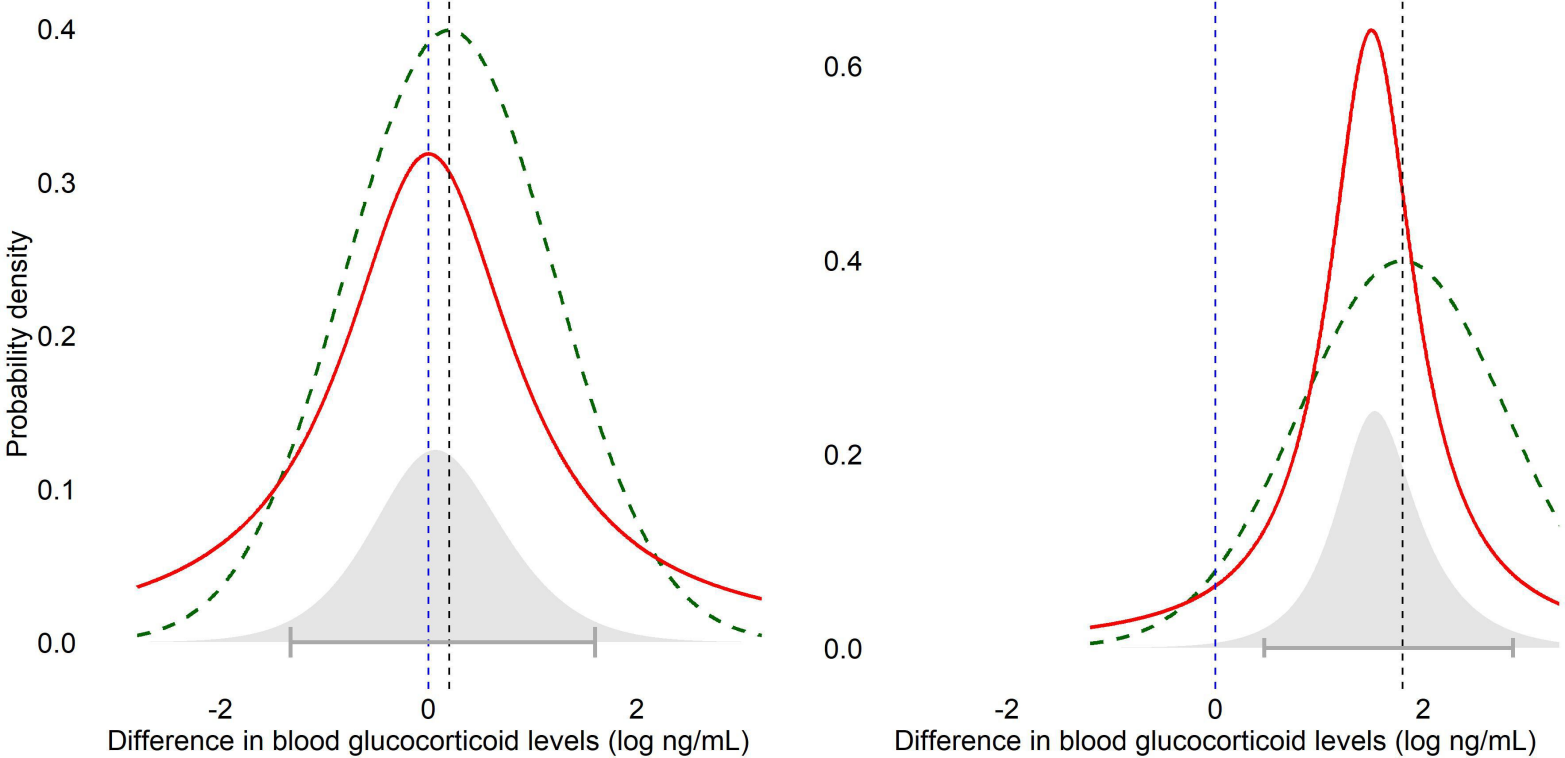
Illustrations of Bayesian statistics applied to investigate differences in the hypothetical blood glucocorticoid levels of laboratory-housed zebra finches chronically exposed to different aviary configurations. The blue dashed line represents the nil null hypothesis. For simplicity, the observed effect size matches the true effect size in both cases, thus the sample data are an accurate reflection of reality. Two outcomes are presented. In the left-hand panel, the true effect size (denoted by the vertical black, dashed line) is log 0.2 ng ml^−1^, and the prior distribution of possible effect sizes under the alternative hypothesis is modelled as a Cauchy distribution centred on 0 and a standard deviation of 1 (red curve). The Bayes factor is 1.89, neither favouring the nil null hypothesis nor the alternative hypotheses, and the 95% credible interval includes 0 and thus also does not favour either hypothesis. In the right-hand panel the true effect size is log 1.8 ng ml^−1^, the prior distribution is centred on 1.5 with a standard deviation of 0.5 due to substantial prior evidence suggesting what the expected effect size should be. The Bayes factor is 0.29, favouring the alternative hypothesis, and the 95% credible interval includes positive values only and therefore also favours the alternative hypothesis. In both cases, the dashed, green curve represents the likelihood of the observed data given the true effect size. The grey shaded area is the marginal likelihood—the distribution of likelihoods across all possible effect sizes, weighted by the distribution of possible effect sizes under the prior distribution of the alternative hypothesis. You can see that the height of the marginal likelihood under the alternative hypothesis (grey shaded area) is lower than the peak of the likelihood of the observed data given the true effect size (dashed, green curve), and this reduction reflects the influence of the broad prior, i.e. averaging over a range of possible effect sizes rather than a single point. The marginal likelihood is used to compare the plausibility of the data under the two hypotheses and does not directly estimate where the true effect size lies—for that you inspect the 95% credible interval associated with the posterior distribution. N.B. If you included prior odds in the analysis, these would be applied after the calculation of the Bayes factor to determine the posterior odds between hypotheses for model comparison. Importantly, prior odds do not affect the 95% credible interval, which is derived solely from the posterior distribution of the effect size.

Related to likelihood ratios are Bayes factors. When exploring evidence for the null, rather than comparing the likelihood of the observed data at two different point values, Bayes factors typically compare the likelihood of the data at 0 (the nil null; again a point value) against a probability distribution of effect sizes under the alternative hypothesis deemed plausible before the observed data, quantifying the relative evidence for the hypotheses as a ratio [20] (figure 3). So, in contrast to likelihood ratios, the Bayes factor incorporates uncertainty about the effect size predicted by the alternative hypothesis by averaging over a range of plausible values, asking how good the whole model is on average compared to the nil null hypothesis [21]. (In other words, likelihood ratios can be considered a special case of Bayes factors where the prior distribution is a point.).

What distribution of plausible effect sizes should the alternative hypothesis represent? The so-called prior distribution should reasonably reflect what you believed was plausible before seeing the data. The most commonly chosen distribution is Cauchy, which is similar to Gaussian and typically centred at 0 [22]. This is the default in the R function BayesFactor() [23], code that works for many experimental designs (a graphical user interface is available on JASP (jasp-stats.org)). This setting for the alternative hypothesis predicts that the effect size is most likely to be near 0 and less likely at values absolutely greater than 0. It is important to note that the parameters of the alternative hypothesis are set not to create a straight comparison with the nil null hypothesis but rather enable a robust analysis of the nil null given newly observed data.

You can include a different prior distribution for the alternative hypothesis into your analysis if you have a specific expectation about the presence of an effect, e.g. the size of the difference between conditions and an estimated standard deviation [20] (figure 3, right-hand panel). Defining the alternative hypothesis, i.e. effect size, for a Bayes factor analysis is not always easy but [24] provides helpful suggestions.

If the Bayes factor is large enough, then your data are sufficiently more likely under the nil null hypothesis than the alternative hypothesis to conclude that there is no effect (again assuming the evidence for the nil null is the numerator in the ratio). Lee & Wagenmakers [25] recommend a Bayes factor of 3 as moderate evidence and 10 as strong evidence in support of the nil null hypothesis. Bayes factors closer to 1 are inconclusive—the data are termed insensitive in distinguishing the alternative hypothesis from the null hypothesis of no effect, and thus you cannot conclude that there is or is not an effect.

It is not possible to easily calculate a Bayes factor for every experimental design. However, as I describe in [26], there is a simple formula you can apply to convert a *p*-value to what is termed the Bayes factor upper bound—the largest Bayes factor in favour of the alternative hypothesis that is reasonably likely, i.e. with the most optimistic but defensible prior distribution, and is calculated simply as:

Bayes factor upper bound ≤ −1/e .p.ln(p) (comment by Benjamin & Berger, annexed to [27]).

All the above applications of the Bayes factor implicitly assume that you believe the two hypotheses to be equally likely; technically this is termed as the prior odds = 1 [22]. However, if you have reason to believe one of the hypotheses to be more likely prior to observing your data, you can recognize this by designating prior odds weighted towards one hypothesis. In this case, the Bayes factor represents the amount that your relative belief in each of the two hypotheses should change from those prior odds. The posterior odds summarize your updated belief about which hypothesis is more likely subsequent to your data collection and are calculated by averaging across your prior beliefs and your data:


Priorodds×BayesFactor=Posteriorodds.

For example, if you consider prior to data collection that the nil null hypothesis is three times more likely than the alternative hypothesis, and you calculate a Bayes factor of 2 based on your data, then while the Bayes factor itself offers very weak evidence for the nil null, the posterior odds indicates that no effect is six times (3 × 2) more likely than the alternative. The calculation to incorporate prior odds is pleasingly simple, but assigning the value of prior odds in the first place may not be. Nonetheless, assigning prior odds can be summarized into two schools of thought: subjective Bayes where the prior odds should reflect your *a priori* beliefs, and objective Bayes where prior odds should reflect as few assumptions as possible [22]. You might lean towards the latter approach if you consider that beliefs are superseded by what can be demonstrated Taper and Lele [28].

## 5. Bayesian estimation: what is the probability of the nil null hypothesis given prior beliefs combined with the subsequently observed data?

Having decided upon a prior distribution regarding the effect size and then observed new data, the posterior distribution can in turn be calculated, which is an update on your beliefs about the likelihood of the effect size. A credible interval can then be calculated from the posterior distribution, which is somewhat analogous to a confidence interval, and indeed the two become numerically more similar as the chosen distribution of the effect size under the alternative hypothesis becomes broader [29, section 4.4] (figure 3). The 95% credible interval represents the central 95% probability mass of the posterior distribution. In other words, you believe there is a 95% probability that the true effect size lies within the credible interval (though with a mis-specified prior distribution your credible interval could be much too small). (N.B. Ninety-five per cent is typically used because of the convention with confidence intervals, but the credible interval width is not related to type 1 error rates; [20].) Similarly to the interpretation of confidence intervals, when the credible interval includes 0 you can conclude that there is not strong evidence against the nil null hypothesis, but in turn non-0 values are also included and so the data are consistent with both a nil and non-nil effect. Thus, to accept the null, you must *a priori* define it as a non-nil null hypothesis—a region of ‘not meaningful values’, whereby if the entirety of the interval is contained within that region you can conclude no meaningful effect. Otherwise, if the credible interval includes both 0 and any values above and/or below your designated region of not meaningful values then you cannot conclude there is a lack of support for the nil null hypothesis or for the null hypothesis of the smallest effect size of interest [24].

## 6. Conclusions

When researchers prioritize publishing their ‘statistically significant’ findings [1], they create bias in the literature [30], distorting what we think we know. One reason researchers are less enthused to publish ‘statistically non-significant’ findings is the perception that null results are an unclear outcome. Wagenmakers argues that the null hypothesis, which anyway we know not to be true, cannot be corroborated by standard *p*-value interpretations, hence the null is never concluded but rather forever remains ‘in a state of suspended disbelief’ [31, p. 795]. Using traditional interpretations of the *p*-value, arguably this is correct—a high *p-*value leaves us in limbo about whether the null is true or our study has not revealed the effect that in fact exists. Perhaps research culture will change if scientists can feel more confident in their ability to determine no effect.

In this short article, I’ve introduced five credible approaches to evidencing lack of an effect or meaningful effect. Let us summarize in what ways, with caution, these approaches support you testing nil and non-nil null hypotheses: equivalence tests and confidence intervals cannot enable us to conclude no effect at all, but they can reject effects as larger than the smallest meaningful effect size; likelihood ratios and Bayes factors cannot tell us which if any hypothesis is correct, but they can provide relative evidence for defined candidate hypotheses; Bayesian estimation credible intervals cannot support the concluding of zero effect, but do encapsulate the values deemed credible given prior beliefs and your observed data, and thus can reject the claim that an effect is meaningful [20].

A final word of caution: a researcher reading deeply about statistics with the honourable aim of better analysing his hard-earned data, risks focusing so much on the philosophies, mathematics and coding of these approaches that upon application he does everything but the most insightful of activities—studying and interpreting plots of his measurements. Statistical tests, no matter how many you have at your fingertips, do not supersede graphing your data. Traditionally, we tend to graph significant results while suppressing the visualization of non-significant results—another manifesation of our pathological disdain for supposedly ‘uninteresting’ null findings. Yet the adage that a picture speaks a thousand words surely pertains to data plots too, and outputs from statistical tests are secondary interrogations of your data [32,33]. Both show and then tell the absence of an effect.

## Data Availability

This article has no additional data.
